# Main group mechanochemistry

**DOI:** 10.3762/bjoc.13.204

**Published:** 2017-10-05

**Authors:** Agota A Gečiauskaitė, Felipe García

**Affiliations:** 1 Division of Chemistry and Biological Chemistry, School of Physical and Mathematical Sciences, 21 Nanyang Link, 637371, Singapore

**Keywords:** ball milling, main group, mechanochemical synthesis, mechanochemistry

## Abstract

Over the past decade, mechanochemistry has emerged as a powerful methodology in the search for sustainable alternatives to conventional solvent-based synthetic routes. Mechanochemistry has already been successfully applied to the synthesis of active pharmaceutical ingredients (APIs), organic compounds, metal oxides, coordination compounds and organometallic complexes. In the main group arena, examples of synthetic mechanochemical methodologies, whilst still relatively sporadic, are on the rise. This short review provides an overview of recent advances and achievements in this area that further validate mechanochemistry as a credible alternative to solution-based methods for the synthesis of main group compounds and frameworks.

## Introduction

The original mainspring for the current expansion of solid state methodologies is the need for cleaner, safer and sustainable chemical transformations – particularly since raw materials are becoming ever scarcer. A straightforward strategy to addressing the above is to simply remove or minimise solvent usage throughout any designated synthetic routes. One way to achieve a solvent-free, or nearly solvent-free, synthetic route is via the use of solid-state mechanochemical methodologies. Mechanochemistry [1–10] is an emerging solid state methodology involving the use of little or no solvent, with the potential to challenge the current dominance of ‘wet’ chemical synthesis [11–15]. From a purely synthetic point of view, it is clear that complete eradication of solvents might not be entirely beneficial. Solvents ameliorate reactant interactions, control reaction rates, and aid heat dispersion in exothermic reactions inter alia. Needless to say, solvents are necessary for the extraction, separation, and purification of the final products and/or reaction intermediates [16], which are not always attainable by solvent-free methods [17]. However, the benefits associated with solvent-free or nearly solvent-free synthetic routes are becoming increasingly difficult to deny [11–13], even in the eyes of the most sceptical synthetic chemist.

Mechanochemistry is defined as the field of reactions caused by mechanochemical forces (e.g., compression, shear or friction) [18–19]. Examples of mechanochemical methods are manual and ball-milling grinding techniques [20–22]. Traditional manual mortar and pestle grinding methods are susceptible to variable factors, both human and environmental [23]. In contrast, modern milling technologies address these issues through the use of enclosed solvent-free reaction environments and well-defined experimental conditions throughout the mechanochemical process [24–25]. Amongst the commercially available ball milling designs [23–24], shaker and planetary mills are the most common mechanochemical apparatuses employed in synthetic laboratories [7,16,26–27].

The energy input may be adjusted by modifying parameters including milling time and frequency. Of equal importance in the reaction design is the choice of milling media (i.e., the milling jar loaded with one or more ball bearings). For example, milling balls made from denser materials (e.g., 2.3 g·cm^−3^ vs 15.6 g·cm^−3^ for Teflon and tungsten carbide, respectively) carry greater kinetic energy during the milling progress. The potential for metal leaching [28], rates of wearing [25], and/or promoting chemical reactions [9,29–33] must also be taken into account when selecting appropriate milling media. In addition to variable mechanical and milling media parameters, an alternative approach to controlling the mechanochemical process is via the use of small amounts of liquid and/or solid additives, termed ion- and liquid-assisted (ILAG) or liquid-assisted grinding (LAG), respectively [34–35]. These techniques, in contrast to “dry” milling, often offer advantages such as shorter reaction times and/or greater product selectivity [36].

**Scheme 1:**
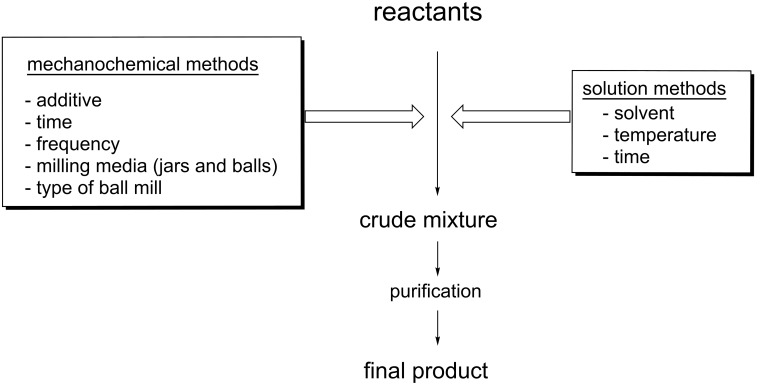
Variables associated with ball-milling (left) and solvent-based methodologies (right).

In traditional solution-based methods, appropriate selection of solvent, temperature and reaction time will determine whether an intended chemical reaction proceeds, to what extent, and the rate at which it does so. In approaching the same chemical reaction by a mechanochemical route, an alternative set of parameters is fine-tuned to optimise reaction conditions (see Scheme 1). Such differences may have the capacity to create unique reactivity patterns and/or access to otherwise unattainable products [11–15].

**Scheme 2:**
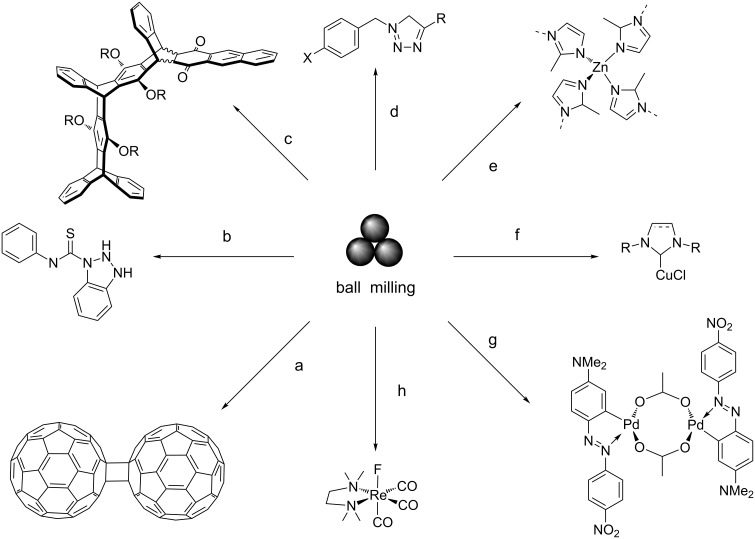
Examples of mechanochemically produced species (a [48], b [62], c [63], d [64], e [65], f [66], g [67], h [68]). The symbol for mechanochemical reactivity proposed by Hanusa et al. [11] is shown centrally.

Although often considered novel, in its broadest terms, mechanochemistry dates back two millennia [20,37–38]. However, it was not until the 19th century that associations between mechanical forces and chemical reactivity were drawn [39–45], and a century later the currently accepted definition of mechanochemistry was proposed [46]. Since then, and during the last 25 years, mechanochemical methods have been applied to various fields, including catalysis [47], organic synthesis [5,7,48–49], metal-organic frameworks (MOFs) [50–51], coordination [52], organometallic [11], supramolecular [53], environmental [54–55], APIs [56], medicinal [57], nanoscience [15], polymer [58–60] and enzymatic chemistry [61]. The recent advances made in mechanochemistry provide an exciting platform for synthetic chemists in the search of novel outcomes and optimal synthetic routes (see Scheme 2).

## Review

### Main group mechanochemistry

Whilst mechanochemical studies have become increasingly popular (with recent reviews on mechanochemical synthesis by Rightmire and Hanusa [11], Do and Friščić [12], Hernández and Bolm [13], Wang [14], and James et al. [2]) there are relatively few studies concerning main group elements.

The development of new and novel main group frameworks and compounds is pivotal to shaping chemistry as a discipline, in addition to advancing neighbouring fields such as biomedical, materials and engineering sciences [69]. Moreover, main group compounds represent a large proportion of all commercial inorganic chemicals (ammonia, silicones, etc.) [70], and recent advances continue to propel the importance of this field in the 21st century [71]. For instance, developments in fundamental main group chemistry are pivotal in providing the necessary knowledge and tools for the more sustainable chemical processes, from “blue-skies” to applied research and, eventually, integration into industrial processes.

Herein, we aim to provide an overview of recent advances in this area and an outlook on future directions within the realm of main group molecular systems.

Developments in the area of materials science have already demonstrated benefits of implementing mechanochemical methods [72]. In the context of main group compounds, recent studies have highlighted efficient routes to: (i) alkaline earth carbides and their intercalation compounds, including the first successful synthesis of Mg_2_C_3_ from its elements [73–74]; (ii) nanomaterials, where main group elements act either as a matrix or a dopant, for catalytic applications [75–76]; and (iii) MOFS containing alkaline earth metals [77–78].

Of particular note are the high yielding syntheses of non-solvated AlH_3_ from LiAlH_4_ and AlCl_3_ under mild conditions, and kinetic studies on the synthesis of alkaline-earth metal amides. These compounds – promising candidates for fuel-cell technologies based on chemically stored hydrogen – highlight the potential of such syntheses in the development of clean energy solutions [79–80].

Within the area of molecular synthesis, the handful of reported examples fall into two general categories, those that provide enhanced synthetic routes, and those that provide novel synthetic outcomes [81].

#### Mechanochemical enhanced synthesis

Optimising the route to a desired reaction product is a principle priority of synthetic chemists, and ball milling often offers attractive opportunities to do so.

**Scheme 3:**
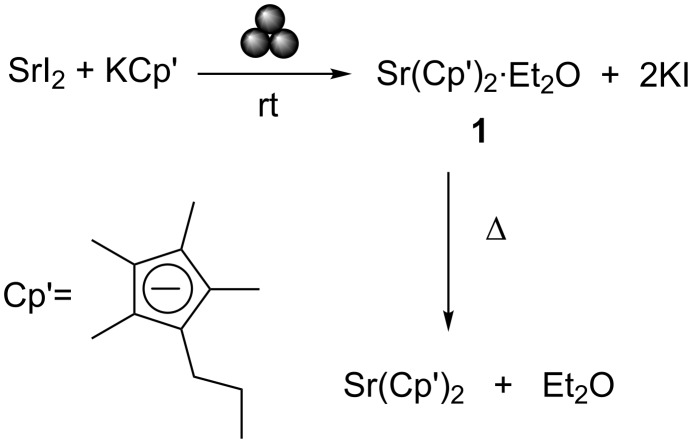
Mechanochemical synthesis of SrCp′_2_(OEt_2_) (Cp′ = C_5_Me_4_(*n*-Pr)).

In the field of organometallic chemistry, we highlight the large-scale synthesis of SrCp′_2_(OEt_2_) (Cp′ = C_5_Me_4_(*n*-Pr)) (**1**) [82], an ideal precursor for the chemical vapour deposition (CVD) of strontium-based semiconductors – a key material in memory devices [83]. Previously, this compound could only be obtained in small-scale via salt metathesis reactions due to poor starting material (SrI_2_ and K[Cp′]) solubility in ether solution. LAG provides a high yielding synthetic methodology circumventing the scalability issues associated with the inefficient diffusion of reactants in large-scale solution-based methods (see Scheme 3).·

**Scheme 4:**
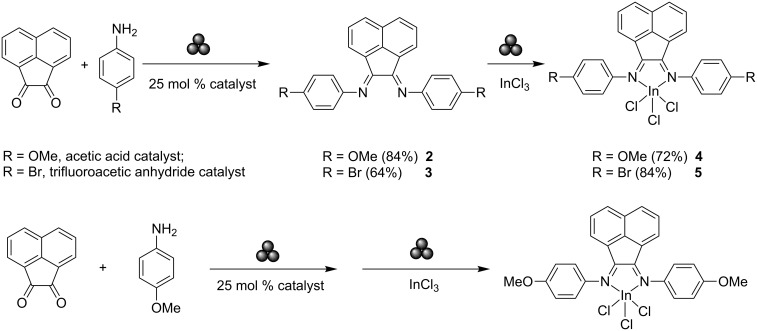
Mechanochemical synthesis of the Ar-BIAN ligands and indium(III) complexes (top). One-pot synthesis of an indium complex (bottom).

Also noteworthy is the multistep solvent-free mechanochemical route to indium(III) complexes featuring aryl bis(imino)acenaphthene (Ar-BIAN) ligands [84]. Ar-BIAN ligands are versatile π-acceptors and have been widely employed for catalysis. These ligands are typically synthesised by condensation reactions between acenaphthoquinone and the corresponding aniline derivative under acidic conditions, involving the use of transition-metal templates [85]. The acid-catalysed ball-milling of acenaphthoquinone with aniline derivatives in the presence of an organic catalyst was able to produce the desired Ar-BIAN ligands **2** and **3**, respectively, in good yields (see Scheme 4). Here, mechanochemistry bypasses the use of templating agent transition metals, shortening the synthetic route and reducing its environmental impact. Their respective indium(III) BIAN complexes **4** and **5** were also obtained by further milling equimolar quantities of the relevant BIAN ligand (**2** and **3**, respectively) and InCl_3_. Performing both reactions at 180 °C in the same reaction vessel without milling lead to thermal decomposition, illustrating the requirement of mechanochemical forces for successful reaction completion. A rare example of a one-pot multistep ball milling reaction is the case of an electron-rich aniline derivative that produced **4** in good yield without the need for ligand isolation. Previously reported examples typically employ “preformed” ligands and metal complexes [68,86], raising the orthogonality of multistep mechanochemical synthesis and widening its applicability.

**Scheme 5:**
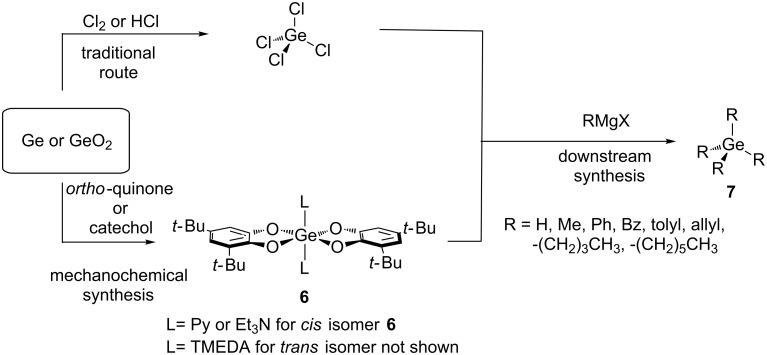
Synthesis of germanes from germanium (Ge) or germanium oxide (GeO_2_).

A significant example of the transformative potential of mechanochemistry is its ability to produce metal complexes directly from bulk metal or metal oxides [66]. Within this context, the LAG synthesis of germanes (GeR_4_) directly from germanium metal or germanium dioxide (GeO_2_) was recently reported [87]. Milling of germanium powder or GeO_2_ with quinone or catechol, respectively, in the presence of a Lewis base under LAG conditions, produced a series of germanium complexes (see Scheme 5). These complexes are inherently versatile, capable of acting as chemical intermediates for the downstream synthesis of germanes **7**, thus providing a sustainable alternative to the use of GeCl_4_. Notably, this method can generate highly pure GeH_4_ for CVD applications under room temperature conditions [88–89].

**Scheme 6:**
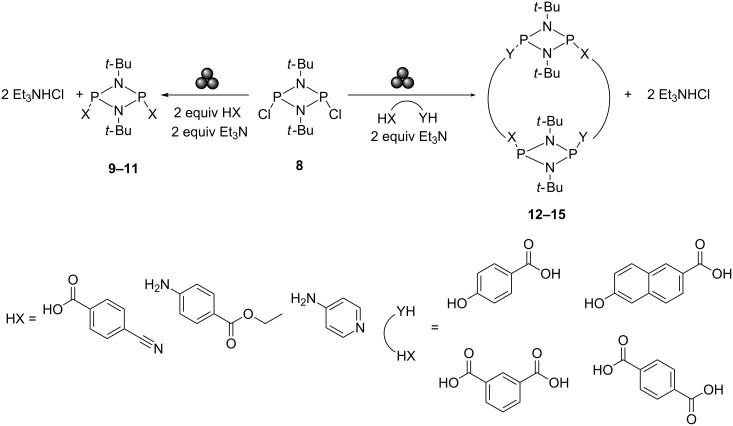
Ball-milling nucleophilic substitution reactions to produce acyclic and cyclic cyclodiphosphazanes.

In addition to organometallics, mechanochemistry has emerged as a technique with great promise for the construction of frameworks based on non-carbon backbones [90], such as those of the phosphazane family [91]. The unique chemical versatility of these P–N frameworks – provided by the diversity of their topological arrangements – provides potential in numerous applications [92]. However, these species remain typically arduous to synthesize and isolate, since phosphazane arrangements are generally highly air- and moisture-sensitive [93–94], and their halogenated precursors are incompatible with protic solvents. Mechanochemistry therefore offers an elegant synthetic route by circumventing solvent compatibility issues, the tedious processes associated with the use of strict anhydrous solvents, and by minimizing unwanted side-products (see Scheme 6) [95].

#### Mechanochemistry picks the lock

The capacity of mechanochemistry to produce unique reaction outcomes and/or product distributions, compared to those obtained by solution-based methods, is an exciting feat [62,96].

Hanusa et al. reported the successful application of ball-milling for the synthesis of an elusive [97] unsolvated tris(allyl)aluminium complex [98]. Grinding 1,3-bis(trimethylsilyl)allylpotassium salt with AlX_3_ (X = Cl, Br, I) produced the desired unsolvated product **16** in high yields and on multigram scales. Remarkably, the synthesis of this long sought-after compound was carried out in a simple set-up, consisting of a round bottom flask loaded with steel ball bearings, connected to a rotatory evaporator as a milling device [99]. Compound **16** displays a higher reactivity than its solvated counterparts, attributed to the coordinatively unsaturated Al centre (i.e., three-coordinate Al). Only in the absence of solvents can this be achieved.

**Scheme 7:**
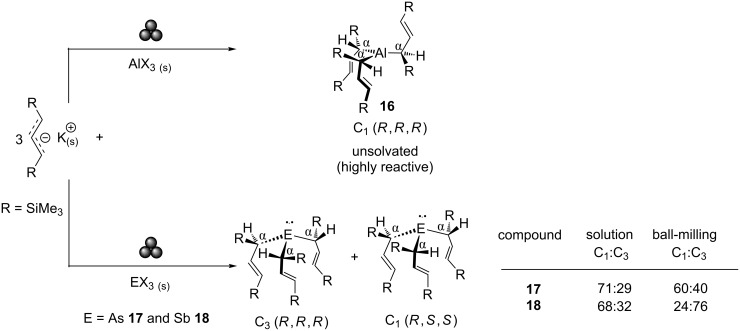
Mechanochemical reactions of potassium 1,3-bis(trimethylsillylallyl) with group 13 (top) and 15 (bottom) halides.

Similar studies using group 15 halides (AsI_3_ and SbCl_3_) have shown that selection of solution or mechanochemical conditions influence product stereoisomer distributions. In this case, the mechanochemical route increases the C_1_:C_3_ stereoisomer ratio in complexes **17** and **18** for As and Sb, respectively (see Scheme 7) [100]. The ability to manipulate isomeric distribution outcomes offers obvious advantages in the application of synthetic mechanochemistry to pharmaceutical and catalysis industries.

**Scheme 8:**
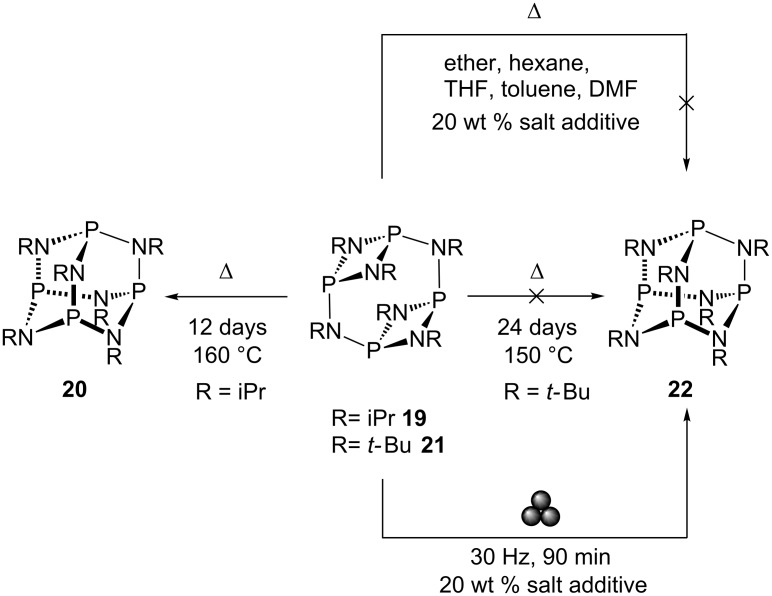
Synthesis of adamantoid phosphazane framework from its double-decker isomer for R = iPr and *t*-Bu (left and right, respectively).

Returning to phosphazane chemistry, the structure of the P–N backbone is controlled by steric factors, a textbook example of which being the adamantoid P_4_(NR)_6_ frameworks. These species are synthesised by direct reaction of PCl_3_ with sterically unhindered amines RNH_2_ (R= Me, Et, iPr, Bz) [101] or by isomerization of their less thermodynamically-stable macrocyclic [{P(μ-NR)}_2_(μ-NR)]_2_ counterparts. In the latter, the isopropyl (iPr) substituted macrocycle [{P(μ-NiPr)_2_}_2_(μ-NiPr)]_2_ **19** readily isomerises into the adamantoid framework, P_4_(NiPr)_6_ **20**, upon heating [102]. The non-viability of the *tert*-butyl (*t*-Bu) substituted adamantoid framework [103] has been rationalised on steric grounds, due to its highly sterically-encumbered nature [103–105]. ILAG milling of [{P(μ-N*t*-Bu)_2_}_2_(μ-N*t*-Bu)]_2_ **21** in the presence of LiCl readily yielded the adamantoid P_4_(N*t*-Bu)_6_ **22** after 90 min, in strong contrast to previous efforts involving prolonged heating (24 days at 150 °C) or under reflux in a range of solvents with identical amounts of salt additive (see Scheme 8). The ease of this transformation by ball milling illustrates the potential of such approaches towards established chemical syntheses [106].

## Conclusion

The use and study of mechanochemical methods have expanded rapidly over the last two decades, and continues to progress as a well-established area of research within chemical and materials sciences. Whilst the synthetic potential of the ball-milling concept has, in our opinion, become indisputable, advancing from representing an anecdotic alternative to solution-based methods, towards becoming a universally adopted methodology by the main group community remains a considerable challenge [69]. Advancement of our current mechanistic understanding of mechanochemical methods is essential if we are to incorporate it as a mainstream tool for synthetic and materials chemists alike [77,107–111]. Theoretical and systematic studies that elucidate the kinetic and thermodynamic driving forces of mechanochemical reactions are undergoing and will be imperative to achieving this goal [106,112–114]. Areas in which we anticipate mechanochemistry will show particular strength in: (i) the synthesis of highly air- and moisture-sensitive compounds, since many are incompatible with a wide range of protic solvents [95]; and (ii) the synthesis of unsolvated species, where chemical reactivity might be hindered by the presence of strongly bound solvent molecules within their coordination sphere [98].

In this short review, we have presented basic underlying concepts followed by recent advances and highlights of mechanochemistry in the context of main group synthesis with the hope of encouraging and accelerating the endorsement of mechanochemistry by the main group and wider synthetic communities.
